# The effect of voice training interventions on patients with oropharyngeal dysphagia: a systematic review

**DOI:** 10.1007/s00405-022-07719-7

**Published:** 2022-11-07

**Authors:** Chunyan Niu, Wenyan Zhou, Haifang Wang, Yingying Zhang, Jianzheng Cai, Nini Lu, Yalan Wang

**Affiliations:** 1 The First Affiliated Hospital of Soochow University, Suzhou, 215006 China; 2 Changshu Second People’s Hospital, Changshu, 215500 China; 3 Department of Nursing, The First Affiliated Hospital of Soochow University, Suzhou, 215006 China; 4 Department of Rehabilitation Medicine, The First Affiliated Hospital of Soochow University, Suzhou, 215006 China

**Keywords:** Voice training interventions, Oropharyngeal dysphagia, Rehabilitation, Swallowing disorders, Therapy effect

## Abstract

**Background:**

Voice training has been proposed as an intervention to improve swallowing function in patients with dysphagia. However, little is known about the effects of voice training on swallowing physiology.

**Objectives:**

This systematic review investigates the effect of voice training on the swallowing function of patients with oropharyngeal dysphagia and provides the theoretical basis for improving the swallowing function and life quality of patients with oropharyngeal dysphagia.

**Data sources:**

A systematic review using a narrative synthesis approach of all published studies was sought with no date restrictions. Five electronic databases (EMBASE, PubMed, CINAHL, Web of Science, and The Cochrane Library) were searched from inception to April 2022.

**Study selection:**

Eight studies were included. Two researchers screened the literature according to inclusion and exclusion criteria, extracted data, and carried out quality control according to the Cochrane handbook5.1.0. Data were analyzed narratively and descriptively.

**Conclusions:**

In general, statistically significant positive therapy effects were found. Voice training improves the oral and pharyngeal stages of swallowing in patients with neurological causes of dysphagia, such as stroke, and in patients with non-neurological causes of dysphagia, such as head and neck cancer. However, the current literature is limited and further primary research is required to provide more evidence to support voice training intervention in dysphagia.  Future studies could  further refine the content of voice training interventions, increase the number of patients enrolled, assess the long-term effects of voice training interventions and add associated assessments of the quality of life after treatment.

## Introduction

Oropharyngeal dysphagia (OPD) refers to the difficulty or inability to move a bolus safely and effectively from the oral cavity to the esophagus, which can lead to related clinical complications, such as malnutrition, dehydration, and severe complications, such as aspiration pneumonia, suffocation, and eventually, premature death. As reported, the global prevalence of OPD was estimated to be 43.8% [1]. Furthermore, the prevalence of OPD is higher with predisposing conditions, such as stroke, Parkinson’s disease and pneumonia [1–3]. Additionally, OPD impacts the quality of life and psychological well-being of patients. Social activities and daily routines are disrupted, resulting in isolation and social exclusion [4].

Patients with OPD represent a large population, and OPD is a daily problem for patients, seriously threatening the average survival time after the deterioration symptoms [5, 6]. Thus, it is highly valued by health workers and their families. Therapeutic approaches to improve the safety and efficiency of swallowing can be divided into compensatory behavioral strategies, dietary modifications, and rehabilitation exercises, most of which are direct interventions for swallowing [7]. Currently, dietary changes and the use of thickeners are the standard treatments, but they are quite expensive, and this practice may reduce the quality of life of patients and does not promote their recovery [8–10]. Therefore, finding a better way to improve the symptoms of patients with OPD has become an urgent global health issue.

Voice training (or vocal exercises) consists of intensive phonation exercises that act under the intrinsic and extrinsic laryngeal muscles to improve the coordination between myoelectric and aerodynamic larynx forces, so that each articulatory element reaches its optimal state and maintains optimal function for an extended period [11, 12]. It was identified that the organs associated with swallowing and speech are structurally and neurologically linked [10]. Through appropriate voice training to promote the closure of the vocal cords while driving the coordinated contraction of cervical swallowing muscles, in addition to strengthen the strength and mobility of the pharyngeal muscles [12], voice training can further stimulate some regional networks of the cerebral cortex. These networks include the auxiliary motor area and anterior cingulate area, which seem to be associated with swallowing movement [13], and thus, their innervation promotes swallowing recovery. Appropriate voice training intervention can further help promote the self-management ability of patients [14]. In short, voice training is a convenient, inexpensive, and highly beneficial method for patients with OPD.

Overall, the present study reveals that improving voice function has a positive impact upon swallowing function. Nevertheless, the physiological mechanism by which voice training results in improved swallowing function in patients with OPD have not been clarified, and there are no reviews that have analyzed this phenomenon in depth. Therefore, we attempted to gain more insight into the changes that occur in swallowing function after voice training. The accepted gold standard for swallowing function assessment is still videofluoroscopy. However, there are many other measures of swallowing function [15], such as the Functional Oral Intake Scale and electromyography, which are also widely used and are reliable measurements of swallowing ability. These additional assessment types remain to be comprehensively summarized. Thus, our research questions were:

1. Which Voice Training Intervention protocols have been used to target improved OPD?
2. Which measures of swallowing physiology have been reported?
3. What additional measures have been used to capture the impact of voice training intervention protocols on swallowing?
4. What are the reported results of voice training intervention protocols?

## Methods

The Preferred Reporting Items for Systematic Reviews and Meta-Analyses (PRISMA) statement [16] was used to guide the development and methodology in the present systematic review.

### Search strategy

We performed a systematic review following the PRISMA guidelines [17]. The articles were selected from the databases PubMed, Embase, Cochrane library, Web of Science, and CINAHL in April 2022 using the following descriptors in English: “voice training,” “voice therapy,” “vocal exercises,” “Voice Treatment,” “singing,” “Whistling,” “Rhythmic Vocalizations,” “Chant,” “dysphagia,” “deglutition,” “swallowing,” and “Oropharyngeal Dysphagia.”

### Inclusion and exclusion criteria

Detailed inclusion and exclusion criteria are outlined in Table 1. We did not limits studies by date; however, we restricted our search to English language publications only. The review was restricted to randomized controlled trials (RTCs), controlled studies, case–control studies, cohort studies, and case series designs. Single case studies were excluded from this review, as we aimed to examine articles using representative samples of a reasonable size. Moreover, editorials and narratives were excluded due to their lack of prospective intervention design.

**Table 1:** Inclusion and exclusion criteria

Categories	Inclusion criteria	Exclusion criteria
Participant characteristics	Adults with a diagnosis of Oropharyngeal dysphagia	Animal studies
Interventions	Voice training intervention	
Comparators	Any other exercise or therapy intervention or no comparators	
Outcomes	Any valid and reliable swallowing function outcome	
Study designs	Randomized controlled trials, controlled studies, case–control studies, cohort studies, and case series designs	Single case studies, editorials, narratives

### Study selection

**Fig. 1:**
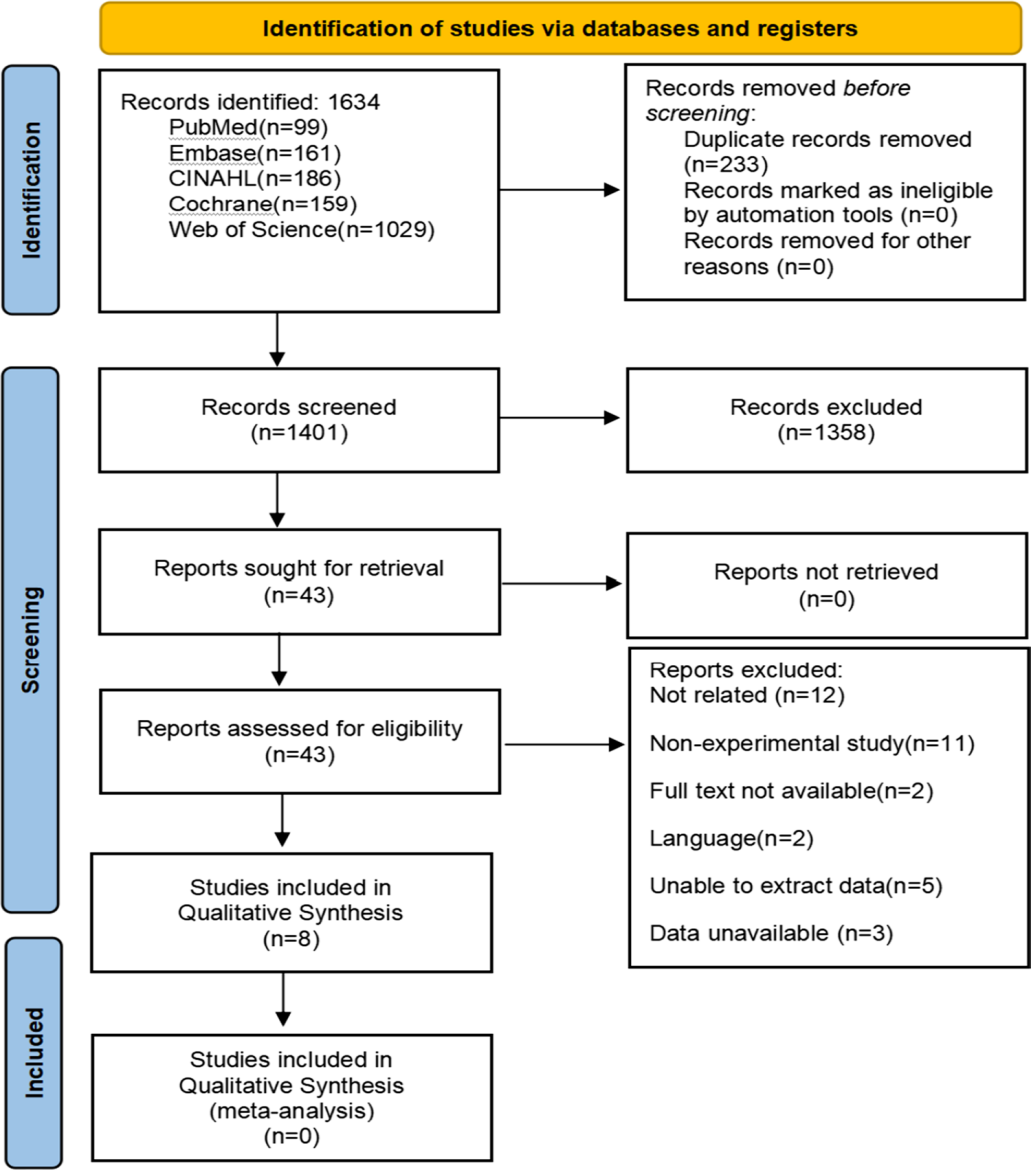
PRISMA flow diagram depicting the different phases of the systematic review, mapping out the number of records identified, included and excluded

As shown in Fig. 1, the original search yielded a total of 1634 records, of which 233 were duplicates. After the removal of these duplicates, two reviewers independently assessed the titles and abstracts of all retrieved records and determined their eligibility for inclusion. Disagreements were settled by consensus and, if needed, by a third reviewer. All reviewers received systematic and relevant training and ensured that they had extensive background knowledge relevant to the research.

### Risk of bias assessment

A methodological quality assessment of each individual study was completed independently by each reviewer to evaluate the validity of the study design and reporting methods. Risk of bias evaluation was completed using the Cochrane Collaboration’s Tool for Assessing Risk of Bias [18]. The criteria assessed were selection bias (random sequence generation, allocation concealment), performance bias (blinding of participants and personnel), detection bias (blinding of outcome assessment), attrition bias (incomplete outcome data), and reporting bias (selective reporting).

Each item on the Cochrane Collaboration’s Tool for Assessing Risk of Bias was scored with a “Y” for yes if susceptible to bias in that category, an “N” for no if not susceptible to bias in that category, or a “U” for unsure/other if raters could not determine appropriate scores, if the criteria were not applicable, or if this was not reported for that particular category.

### Data extraction process

Data extraction was completed independently by a single rater for full articles that met all inclusion criteria outlined above. Data extraction included the following: (1) study design; (2) patient population descriptions (age, sex, etiology); (3) sample size; (4) proportion of male and female participants; (5) interventions details; and (6) outcome measure. Results from each study were extracted and always included statistical analyses of changes in swallowing function after the intervention.

## Results

### Literature retrieval

Figure 1 provides an overview of the selection process for included studies. Of the 1401 studies identified for preliminary screening of titles and abstracts, 1358 were rejected after failing to meet inclusion criteria. After an initial screening of the studies that were considered potentially relevant (43 articles), a full-text reading was carried out, paying special attention to the study design, the intervention (treatment type and outcome evaluation indicators), and other factors. Twelve full-text articles were excluded, as they did not mention voice training interventions for OPD; 11 articles were non-experimental studies; 2 articles were not available; 2 articles were published in English; data could not be extracted from 3 articles, while five studies had a lack of available data. Overall, eight articles met this review’s objective and inclusion criteria [6, 19–25].

### Quality assessment

**Fig. 2:**
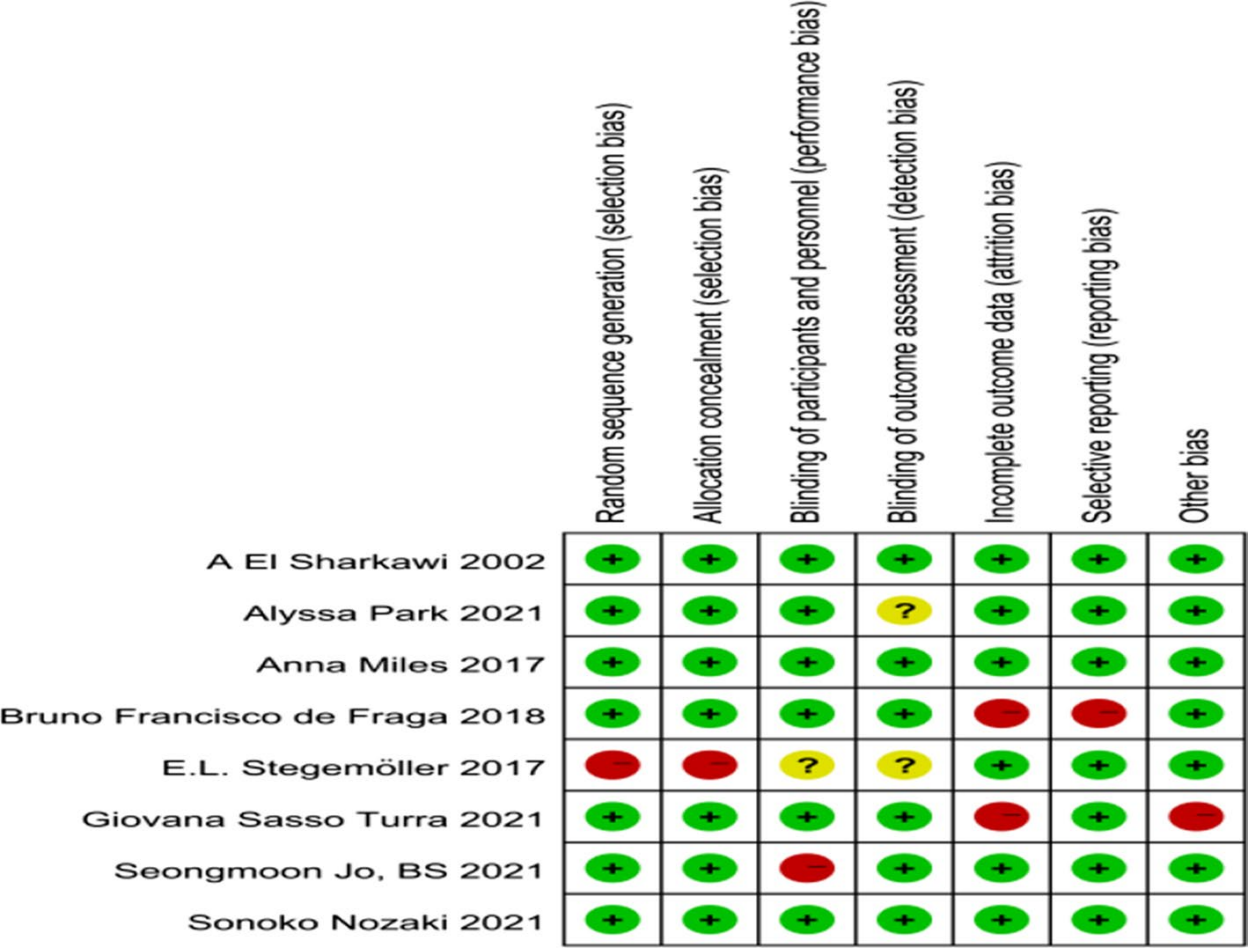
Cochrane tool for risk of bias

Figure 2 summarizes the quality assessment of all included studies using the Cochrane Risk of Bias Tool. Selection bias was identified in one study [6], where participants were enrolled either via convenience sampling or by consecutive recruitment without randomization. Performance bias was identified in two studies. One study [19] was deemed to have a high risk of performance bias as the blindness of study participants was not strictly implemented to the grouping information that was disclosed. One study did not report whether participants and staff were blinded, so it is not certain whether outcomes would have been affected [6]. For two studies, we could not be certain of the examination bias, because there was no mention of blinds in either of the studies by raters of any outcome measures [6, 20]. Two studies were deemed to have a high risk of attrition bias. Participants do not complete the full intervention in one [21] study, and the it is not explained how the interruption data was managed in another study [22]. Reporting bias was deemed to be highly likely in one study [21], which did not report the outcome of a study indicator. Finally, additional biases were identified in one study [22], wherein baseline data varied significantly.

### Patient characteristics

Patient characteristics are reported in Table 2. Eight different patient population groups were included: Parkinson (idiopathic Parkinson) [6, 23, 24], progressive supranuclear palsy [25], head and neck cancer [19], post-orotracheal intubation [22], multiple system atrophy [20], and stroke [21]. Sample sizes varied widely across studies, ranging from 7 participants [25] to 32 participants [22]. Studies included both male and female participants; however, most studies included a larger proportion of males compared to females. The participants were generally older.

**Table 2:** Patient characteristics

Study	Year	*N* (M, F)	Average age in years (SD)	Control group	Etiology
Miles et al. [23]	2017	20 (14, 6)	68 (3.5)	NO	Parkinson
Nozaki et al. [25]	2021	7 (5, 2)	77 (NR)	*N*O	Progressive supranuclear Palsy
Jo et al. [19]	2021	TG13 (10, 3) CG15 (9, 6)	TG 59.15 (4.22) CG 50.87 (3.68)	*N* = 15	Head and neck Cancer
Turra et al. [22]	2021	TG15 (10, 5) CG17 (3, 14)	TG 74 (NR) CG 59 (NR)	*N* = 17	Post-orotracheal intubation
Park et al. [20]	2021	13 (NR)	66.69 (6.58)	NO	IPD MSA
Fraga et al. [21]	2018	TG 5 (3, 2) CG5 (3, 2)	TG 63.8 (12.9) CG73.2 (7.6)	*N* = 5	Stroke
Stegemöller [6]	2017	LD18 (6, 12) HD6 (2, 4)	LD69 (7) HD65 (11)	NO	Idiopathic Parkinson
El Sharkawi [24]	2002	8 (6, 2)	65.5 (10.4)	NO	Idiopathic Parkinson

*N* sample size, *M* male, *F* female, *TG* Treated Group, *CG* Control group, *SD* Standard Deviation, *IPD* idiopathic Parkinson’s disease, *MSA* Multiple System Atrophy, *NR* not reported, *LD* Low dosage (Participants completed one session each week), *HD* High dosage (Participants completed two sessions each week)

### Question 1: training protocols

Voice training interventions included Lee Silverman voice treatment (LSVT), therapeutic singing, and vocal exercises. At present, voice training interventions for patients with OPD focus on LSVT, which was used in 50% of the studies [20, 23–25]. LSVT practices include maximum duration of sustained vowel phonation, maximum fundamental frequency range, and maximum functional speech loudness drill [24]. Therapeutic singing consisted of physical preparation, vocalization for warm-up, singing exercises for laryngeal elevation, and modified singing of approximately 20 min in duration [19]. Vocal exercises include intensive phonation exercises and laryngeal raising and lowering exercises [21]. The most stable duration for LSVT treatment is 4 weeks. The shortest duration of voice training was no more than 10 days. Therapeutic singing practice had a maximum duration of 8 weeks. The duration of each exercise session for the patients in these studies was divided into two types, one of 20–30 min per session and the other of 50–60 min per session; meanwhile, the frequency of weekly voice therapy used in the studies examined spanned a wide range. Exercises were completed with direct clinical guidance from professional language professors or therapists (see Table 3).

**Table 3:** Training protocols

Study	Exercise	Duration (each session)	Frequency (days/week)	Total duration	Guidance
Miles et al. [23]	LSVT	NR	4	4 weeks	Direct supervision by a licensed SLP or authors
Nozaki et al. [25]	LSVT	50–60 min	7	4 weeks	Direct supervision by an LSVT-certified SLHT
Jo S et al. [19]	Therapeutic Singing	20 min	3	4 weeks	Direct supervision by therapists
Turra et al. [22]	Vocal exercises	30 min	7	10 days	Direct supervision by a licensed SLP
Park et al. [20]	LSVT	5–10 min (untreated days 10–15 min)	4	1 month	Direct supervision by a licensed SLP
Fraga et al. [21]	Vocal exercises	NR	7	8 days	Direct supervision by a Researcher team
Stegemöller EL [6]	Therapeutic Singing	60 min (30 min)	1/2 (2)	8 weeks (8 weeks)	Direct supervision by board-certified music therapists (the at-home practice was self-reported)
El Sharkawi [24]	LSVT	50–60 min	4	4 weeks	NR

*LSVT* Lee Silverman Voice Treatment, *SLP* Speech-Language Pathologist, *SLHT* Speech-Language-Hearing Therapist, *NR* not reported

### Question 2: physiological measurements of swallowing

Each study, along with its inclusion criteria, study design, and a list of the outcome measures collected, is presented in Table 4. Of these studies, three used VFSS as the measurement tool. In the literature, fiber-optic endoscopy and/or videofluoroscopy of swallowing are used as the gold standard [26, 27]. All studies use temporal measurements to reflect changes in swallowing.

**Table 4:** Swallowing measures

Study	Inclusion criteria	Study design	Outcome measures collected
Miles et al. [23]	(1) Confirmed Parkinson's patients with voice deterioration; (2) Adequate motivation, cognition and hearing, and logistic ability to attend the full program; (3) A laryngologist assessment reporting no contradictions to treatment	Prospective non-randomized single-blinded cohort intervention study	VFSS (Oropharyngeal measures of timing, displacement and area) EAT-10
Nozaki et al. [25]	(1) Diagnosed with dysphagia or dysarthria; (2) MMSE score > 20; (3) No head or neck cancer; (4) No other neurological disorders that may affect swallowing	Prospective Cohort Intervention Study	VFSS OTD PTD DTOUES
Jo S et al. [19]	Diagnosed with HNC; Underwent surgical procedures; (3) Underwent several sessions of radiotherapy for their tumors	Prospective, randomized, controlled, double-blind trial	VDS Oral phase Pharyngeal phase Total score DIGEST Safety grade Efficiency grade Total grade
Turra et al. [22]	(1) Being hospitalized; (2) 18 years of age or older; (3) Being clinically stable; (4) No neurological diseases; (5) Receiving orotracheal intubation for at least 48 h	Prospective, randomized, controlled, double-blind trial	FOIS (Progression of Oral Intake Outcome) PARD (Severity of Dysphagia Outcome)
Park et al. [20]	(1) Diagnosis of IPD or MSA-C by a neurologist or physiatrist; (2) Complaint of difficulty in swallowing	Prospective Cohort Intervention Study	VDS Oral phase Pharyngeal phase Total score NIH-SSS/SWAL-QOL
Fraga et al. [21]	(1) 18 years of age or older; (2) Diagnosed with stroke; (3) Signing the terms of free and informed consent; (4) No other associated neurological pathologies; (5) Has not undergone previous speech therapy rehabilitation intervention or perform the therapeutic exercises	Prospective, randomized, controlled, double-blind trial	FOIS
Stegemöller EL [6]	Diagnosed with IPD; No smoking; No untreated hypertension; No history of head or neck cancer; No significant cognitive impairment (MMSE score < 24); No major psychiatric disorder (BDI score < 18)	Prospective non-randomized Cohort Intervention Study	UPDRS Total UPDRS; Motor UPDRS UPDRS Neck Rigidity Score UPDRS Swallow Score EMG (submental and laryngeal muscle groups; the THIN and THICK conditions) Peak amplitude AUC Rise time Fall time Duration SWAL-QOL
El Sharkawi A [24]	Diagnosed with IPD; No history of gastrointestinal disease; No gastro-oesophageal surgery; No head and neck cancer; No other neurological disorders that may affect swallowing	Prospective Cohort Intervention Study	VFSS (identification of physiological motility disorders in the oropharyngeal swallow and temporal measures of the oropharyngeal swallow)

*MMSE* Mini Mental State Exam score, *BDI* Beck Depression Inventory score, *VFSS* Videofuroscopic Swallowing Study, *OTD* Oral Transit Duration, *PTD* Pharyngeal Transit Duration, *DTOUES* Duration to the opening of the UES, *VDS* Videofluoroscopic dysphagia scale, *DIGEST* dynamic imaging grade of swallowing toxicity, *NIH-SSS* the National Institutes of Health-swallowing safety scale, *FOIS* Functional Oral Intake Scale, *PARD* Protocolo de Avaliação do Risco para Disfagia, *EAT-10* the Eating Assessment Tool-10, *SWAL-QOL* the Quality of Life in Swallowing Disorders Questionnaire, *UPDRS* the Unified Parkinson’s Disease Rating Scale, *EMG* Electromyography, *AUC* area under the curve

Two studies used the Videofluoroscopic Dysphagia Scale (VDS) as the tool of measurement [19, 20]. VDS has 14 items with a total score of 100, which are divided into the oral phase (7 items, 40 points) and the pharyngeal phase (7 items, 60 points). The higher the score, the more severe the swallowing difficulty [20]. One of the studies did not report scores for each parameter but instead reported total scores for all parameters [20].

One study [6] used electromyography (EMG) to measure swallowing in patients. In previous studies, EMG has been used to describe the swallowing function of patients and is reported to be effective in identifying differences between patients with swallowing disorders and healthy patients [28]. The indicators of swallowing in EMG are Peak amplitude, the area under the curve (AUC), time to peak amplitude, rise time, fall time, and duration [28].

### Question 3: additional measures

Additional measures used to determine the effects of voice training included the Speech Handicap Index-15 (SHI-15), which assesses the effects of patient speech on daily life [29], and the Eating Assessment Tool-10 (EAT-10), which documents the initial dysphagia severity and monitors the treatment response in persons with a wide array of swallowing disorders [30]. Moreover, the Quality of Life in Swallowing Disorders Questionnaire (SWAL-QOL) is used to quantify changes in swallowing related quality of life [31], and the Unified Parkinson’s Disease Rating Scale (UPDRS) is employed to measure the patient's neck stiffness and the effect of swallowing therapy (the patient’s neck stiffness may affect swallowing and electromyography measurements) [24].

In addition, two studies [21, 22] did not use fibroendoscopy or video fluoroscopy to evaluate changes in swallowing before and after treatment, instead opting to use only qualitative scales. Both such studies used the Functional Oral Intake Scale (FOIS), which has been described as an important and reliable tool for assessing oral intake progression [32], to assess changes in functional oral intake [33]. One study used the Protocolo de Avaliação do Risco para Disfagia (PARD) method to characterize clinical signs that are suggestive of laryngeal penetration or aspiration and the severity of dysphagia [34].

### Question 4: voice training intervention outcomes

VFSS measurements: The most commonly collected outcome measures of swallowing physiology to determine changes pre- and post-treatment were temporal measurements [35]. Statistically significant changes in swallowing timing measurements were identified in two [23, 25] studies. Both studies revealed an increase in esophageal sphincter opening time in patients by LSVT. Significant changes in OTT and OPSE were identified in a second study [24].

VDS measurements: Statistically significant changes in pharyngeal phase scores were identified in two [19, 20] studies. In one of the articles [20], an increase in total VDS score was also identified as being statistically significant; more notably, changes in the pharyngeal stage of VDS continued into the follow-up period in this study.

EMG measurements: The results of one preliminary study [6] revealed that EMG time measurements of the laryngeal and subchinnabular muscle groups during swallowing after therapeutic singing were significantly increased in patients with Parkinson’s disease. Moreover, analysis of EMG results revealed no relationship between the swallowing ability of patients and the rate of weekly treatment reviews.

FOIS measurements: Both studies [21, 22] using FOIS demonstrated statistically significant improvements in swallowing before and after treatment.

Other measurements: The results of these measures all revealed improvements in swallowing function in different ways. Detailed information regarding all reported outcomes is provided in Table 5.

**Table 5:** Summary of outcome measures and results

Study	Measures	Results
Miles et al. [23]	VFSS (Oropharyngeal measures of timing, displacement, and area)	Significant improvement in AEdur, PCR and PESmax (*p* < 0.05)
	EAT-10	Statistically significant differences reported when comparing pre-LSVT LOUD and one week and six months post-LSVT LOUD (p < 0.001)
Nozaki et al. [25]	VFSS (OTD, PTD, DTOUES)	Significant improvement in DTOUES reported (p = 0.016)
Jo S et al. [19]	VDS (oral phase; pharyngeal phase; total score)	Significant improvement in swallowing function in the intervention group (*p* = 0.008), especially in the pharyngeal phase, with significant reverse patterns demonstrated in the control group (*p* = 0.042)
	DIGEST (Safety grade; Efficiency grade; Total grade)	Statistically significant differences in both the safety grade (*p* = 0.016) and the total grade (*p* = 0.008) of the intervention group. No significant differences identified in the control group in the safety grade or the total grade Significant improvement in efficiency grade (*p* = 0.006) in the intervention group, with significant reverse patterns in the control group (*p* = 0.025)
Turra et al. [22]	FOIS (Progression of Oral Intake Outcome)	Statistically significant differences reported between the control group and the treated groups (*p* = 0.005)
	PARD (Severity of Dysphagia Outcome)	Significant improvement in PARD (*p* < 0.001) observed in the treated group
Park et al. [20]	VDS (oral phase; pharyngeal phase; total score)	Significant improvement identified in the VDS pharyngeal score in the IPD group when comparing pre- and post-treatment assessment and between pre- and follow-up assessments (*p* < 0.05; *p* < 0.05) Significant improvement in the VDS pharyngeal score and total score of the MSA-C group when comparing pre- and post-treatment assessments (*p* < 0.05)
	NIH-SSS	Significant improvements in the NIH-SSS pre- vs post-intervention for both groups (*p* = 0.046)
	SWAL–QOL	Significant improvement reported in Symptom frequency (*p* = 0.037) and total scores (*p* = 0.039) in SWAL-QOL of the MSA-C group before and after treatment Significant improvement in the eating duration (*p* = 0.012) in SWAL-QOL of the MSA-C group when comparing pre-treatment and the follow-up assessment
	SHI-15	Significant improvement in the SHI-15 psychosocial score in the IPD group when comparing pre- and post-treatment assessment and between pre- and follow-up assessment (*p* = 0.045; *p* = 0.025, respectively) Significant improvement reported in the SHI-15 psychosocial score (*p* = 0.042) in the MSA-C group when comparing pre- and post-treatment assessment
Fraga et al. [21]	FOIS	Statistically significant improvement identified post-therapy in the experimental group (*p* = 0.039). No statistically significant difference reported when comparing between both groups post-therapy (*p* = 0.126)
Stegemöller EL [6]	UPDRS	Significant decrease reported in the total UPDRS and motor UPDRS scores (*p* < 0.001; *p* < 0.001) when comparing the two groups after the intervention Significant increase in the UPDRS neck rigidity score (*p* = 0.03) identified when comparing between the two groups after the intervention
	EMG	The submental muscle group: the THICK condition: Statistically significant alterations in AUC (*p* = 0.04) after the intervention. Statistically significant alteration in peak amplitude reported between the two groups (*p* = 0.03), and after the intervention (*p* = 0.04) The laryngeal muscle group: statistically significant changes reported in rise time (THIN: *p* = 0.02 and THICK: *p* = 0.01), fall time (THIN: *p* = 0.001 and THICK: *p* = 0.001) and EMG duration (THIN: *p* < 0.001 and THICK: *p* < 0.001) after the intervention The submental muscle group: statistically significant in changes in fall time when comparing the two groups (THICK: *p* = 0.01) after the intervention (THIN: *p* = 0.04; THICK: *p* = 0.01); and significant changes in EMG duration (THIN: *p* < 0.001; THICK: *p* = 0.004) after the intervention
	SWAL-QOL	No significant changes reported
El Sharkawi A [24]	VFSS	3 ml liquid bolus: significant reduction in OTT (*p* < 0.05) 3 and 5 ml liquid: significant improvement in oral residue percentage (*p* < 0.05) cup drinking: significant improvement in OPSE (*p* < 0.05)

*AEdur* Airway closure duration, *PCR* pharyngeal constriction ratio, *PESmax* maximal opening of the pharyngoesophageal sphincter, *OTD* Oral Transit Duration, *PTD* Pharyngeal Transit Duration, *DTOUES* Duration to opening of the upper esophageal sphincter, *OTT* oral transit time, *OPSE* oropharyngeal swallow efficiency

## Discussion

### Methodological comments

The review presented herein identified mixed evidence to reveal whether voice training intervention specifically impacts swallowing function. Overall, voice training can improve swallowing in patients with neurological dysphagia, such as stroke, and in patients with non-neurological dysphagia, such as head and neck cancer. For patients with swallowing disorders who suffer from expensive treatments, complex therapies and additional treatment time [36], voice training to improve swallowing function can certainly help alleviate these burdens.

Nevertheless, all studies provided information regarding the short-term effects of the treatment, while minimal data were reported regarding long-term effects. Only two studies conducted follow-ups; however, the longest follow-up period did not exceed 6 months. Therefore, it was difficult to judge the long-term effects of voice training intervention on swallowing function. In addition, 50% of reviewed study protocols did not include a control group, which may result in the misinterpretation of data confounded by the natural recovery over time of oropharyngeal swallowing function in dysphagia patients.

In the field of voice therapy, quality-of-life assessment is already established as an important evaluation technique [37, 38]. However, in this systematic review, this issue was identified as being regularly ignored when describing therapy outcomes. Only one of the final eight studies highlighted quality-of-life issues related to OPD. It is hoped that future studies will value QOL assessments.

Moreover, the evaluation of treatment outcomes in the reviewed studies was broadly limited to a small sample size measured by a small number of speech therapists. In addition, there was large heterogeneity in the patient populations selected in the included articles. Two studies [21, 22] were limited to the use of self-assessment tools only and did not use instrumental evaluations of swallowing to determine the impact of voice interventions on dysphagia. Both of these limitations significantly limit both our understanding in this area and the interpretation of evidence currently available. There are a wide variety of heterogenous methods available for the measurement of swallowing function. It is, however, recommended that objective swallowing measurement tools are utilized, such as VFSS, EMG, etc.; these methods should enable proper assessment of a patient's swallowing function.

Finally, the current voice intervention therapies are relatively simple and applied general; more individualized therapies have not yet been developed for the distinct stages of dysphagia or associated muscle groups. Although, surface EMG can be used to quantify the muscle strength associated with swallowing and also to monitor the status of different muscle groups during pronunciation [15]. A more precise and stratified intervention plan can be constructed by assessing the electromyographic activity of the swallowing surface muscles through the surface EMG technique, which would be beneficial for the rehabilitation of swallowing function in patients.

### Therapy effect comments

In the included articles, patients exhibited improvement in both the oral and pharyngeal phases of dysphagia. During the oral phases, voice training intervention was effective in improving tongue strength, enabling it to better control the bolus and ameliorate premature bolus loss [20, 24]. Improvement in oral transit time (OTT) and premature bolus loss in patients could indicate improved tongue motor function during the oral phases (Table 5). During the pharyngeal phases, vocal training intervention was effective in modifying glottal closure and laryngeal elevation, and these mechanisms were observed to be associated with airway protection. The improvement of maximal opening of the pharyngoesophageal sphincter (PESmax) by VFSS, the improvement of the rising and falling times of laryngeal muscle groups by EMG, in addition to the improvement of VDS pharyngeal phase score, FOIS score, and DIGEST score [6, 19, 21, 22, 22, 23], all indicate improvements in laryngeal muscle swallowing function in patients (Table 5).

Voice training intervention can improve maximum phonation time (MPT) in dysphagia patients [20]. MPT was correlated with oropharyngeal motor functions, such as tongue movement (bolus formation, oral transit time), laryngeal elevation, and pharyngeal swallow triggering [39]. Thus, MPT status may contribute to the mechanisms by which voluntary swallowing is improved.

Because the voice training intervention enhanced swallowing protocol was designed to make patients sing at different pitches, this particular therapy facilitated an increase in the width of the upper esophageal sphincter. Therefore, the therapy resulted in increased the extent and duration of laryngeal elevation [19]. Maintaining elevation of the laryngeal complex and thus the hyoid bone allows the esophageal port to stay open longer, facilitating increased time for the bolus to clear and reducing the chance of aspiration [40]. Voice intervention training is capable of altering the neurophysiologic mechanisms responsible for the upper digestive system, with orofacial myofunctional adjustment aiding the elimination of laryngotracheal aspiration risks [22]. In addition, vocal exercises enable a significant increase in the vibration and movement of the laryngeal and pharyngeal structures, primarily focused at the vocal folds, which leads to an increase in the amplitude of vibrations at the mucous membrane. This increase in vibration facilitates the activity of the inward process, which subsequently improves vocal glottal closure function [21]. In conclusion, both the promotion of laryngeal elevation and vocal fold closure function demonstrates that voice intervention training can improve swallowing function in OPD patients.

Furthermore, some studies identified that in addition to the primary sensorimotor cortex (pharynx–larynx representation) and brainstem, the additional region most strongly activated during voluntary swallowing was the right anterior insular cortex [41, 42]; this region is one of the sites associated with significant change after voice training intervention [43]. Therefore, the right anterior insular cortex may further contribute to the mechanism of improved voluntary swallowing in dysphagia patients [24].

### Limitations

Limited study design, poor methodological quality and the small sample size of included studies may limit the conclusions drawn from our analyses. There may be eligible studies archived in databases and search algorithms that we did not use for the literature search and thus were not identified. Finally, as limited translational resources were available, only English studies were included in this review. However, despite this limitation, only two non-English studies were excluded at the full-text screening stage.

## Conclusions

Overall, this systematic review described the effects of voice training interventions on swallowing function. Voice training improves the oral and pharyngeal stages of swallowing in patients with neurological causes of dysphagia, such as stroke, and in patients with non-neurological causes of dysphagia, such as head and neck cancer. However, the number of included studies was small and they are diverse in terms of assessment tools and cannot be quantitatively analyzed. Therefore, further preliminary studies are now needed to provide more evidence to support voice training intervention in dysphagia.

Currently, voice interventions are available for people with a variety of underlying conditions that cause dysphagia. Future studies should endeavor to further increase the number of patients included, expand the coverage of the treatment population, assess the long-term effects of voice training interventions, increase the evaluation of the improvement in quality of life of patients after swallowing, and provide stronger evidence to deconvolute the effect of speech training on improving swallowing function. Future research should further attempt to refine and stratify the content of speech training with the support of clinical practice to facilitate more rapid swallowing function amelioration in patients.

## Data Availability

The data that support the findings of this study are available from the corresponding author upon reasonable request.
